# Enhancing patient acceptance of ICD implantation through structured shared decision making: conversation is key

**DOI:** 10.1007/s10840-024-01850-7

**Published:** 2024-07-13

**Authors:** Litsa K. Lambrakos, Suzanne A. Feigofsky, Ying Wang, Fozia Z. Ahmed, Marta Pachón, Theodore S. Takata, Camille G. Frazier-Mills, Emily Kotschet, Laura M. Gravelin, Jonathan C. Hsu

**Affiliations:** 1 Division of Cardiology, Department of Medicine, University of Miami Miller School of Medicine, 1321 NW 14th Street, Suite 510, Miami, FL 33136 USA; 2 Iowa Heart Center, Carroll, IA USA; 3 Ipsos, Chicago, IL USA; 4 Keele Cardiovascular Research Group, Keele University, Keele, UK; 5 Cardiology Department, Hospital Virgen de La Salud, Toledo, Spain; 6 Texas Health Heart and Vascular Specialists, Fort Worth, TX USA; 7 Division of Cardiology, Duke University Medical Center and Duke Clinical Research Institute, Durham, NC USA; 8 Monash Cardiac Rhythm Management Department, Monash Medical Centre, MonashHeartMelbourne, VIC Australia; 9 Mount Carmel Medical Group, Columbus, OH USA; 10 Division of Cardiovascular Medicine, Department of Medicine, University of California, San Diego, CA USA

**Keywords:** Defibrillator, Patient-physician conversation, Device acceptance, Artificial intelligence, Informed decision-making

## Abstract

**Background:**

Implantable cardioverter-defibrillators (ICDs) and cardiac resynchronization therapy defibrillators (CRT-D) are lifesaving treatments for patients at risk for sudden cardiac death. Effective physician–patient communication during the shared decision-making process is essential. Electrophysiologist-patient conversations were targeted to obtain objective data on the interaction, understand the conversation framework, and uncover opportunities for improved communication.

**Methods:**

Individuals previously identified as requiring an ICD/CRT-D but declined implantation were recruited for this four-stage interview and survey-based study. Quantitative analysis of surveys and AI analysis of conversation videos was conducted to evaluate patient participant expectations, analyze feedback about the conversations with study physicians, and gauge willingness for device implantation.

**Results:**

The study included 27 patients (mean age 51 years, 51.9% female) and 9 study physicians. Patients were significantly more willing to undergo ICD/CRT-D implantation after conversing with study physicians compared to their own physicians and pre-conversation surveys (mean scores: 5.0, 3.1, and 4.4 out of 7, respectively; *p* < 0.001). Patient participants had higher satisfaction with the study conversation, rating study physicians higher in effectiveness of explanations, responsiveness to questions, and overall quality of the conversation compared to their own physicians (all *p* < 0.001).

**Conclusions:**

In a cohort of patients who previously declined ICD/CRT-D implantation, patient satisfaction and willingness to undergo implantation of a guideline-directed device therapy increased significantly following a structured conversation with study physicians. Identified key elements could be integrated into user-friendly tools and educational materials to facilitate these conversations, improving patient engagement with the decision-making process and enhancing informed acceptance of indicated device therapies.

**Graphical abstract:**

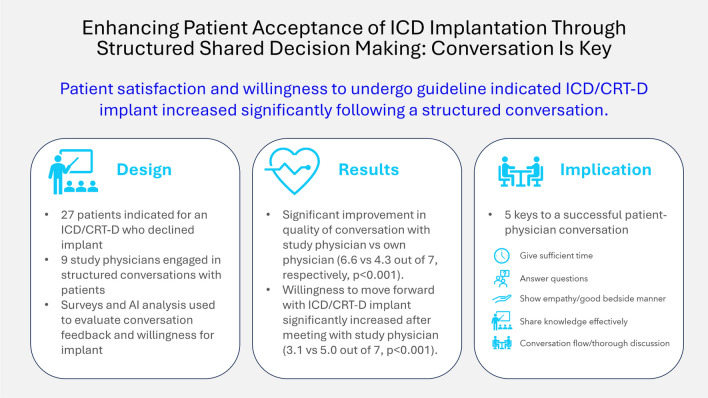

## Introduction

Implantable cardioverter-defibrillators (ICDs) and cardiac resynchronization therapy with defibrillators (CRT-Ds) are lifesaving treatments for individuals at risk for sudden cardiac death and heart failure. Globally, approximately 1.4 million cardiovascular implantable electronic devices (CIEDs) are implanted each year [1]. However, despite the benefits, up to 1 in 6 people with an indication refuse implantation [2]. and overall, utilization of device therapy is low—in a modern cohort of HF patients, appropriate guideline-directed device therapy was used in only 41% of patients indicated for CRT-D and in 14.7% of ICD-indicated patients [3]. Additionally, despite the established benefits of CIEDs, there can be time-dependent (i.e., short vs long term) complications and risks involved. Therefore, the shared decision-making process between patient and physician necessitates thorough information dissemination, including a discussion of anticipated risks and benefits associated with lifelong device therapy, and the elicitation of patient values and preferences [4]. Clinicians, particularly electrophysiologists, bear the ethical responsibility of ensuring that this exchange of information occurs in a manner that is conducive to a patient-level understanding of the process for informed consent. Research indicates that patient satisfaction with information delivery in a clinical context is correlated with quality of life related to that therapy [5].

However, recent studies have demonstrated that there are major gaps in the information provided to patients at the time of CIED implantation [4]. Patients’ desire for more knowledge resources and a better understanding of their CIEDS is not always fulfilled by the patient-physician consultation [4, 6]. A European survey of ICD recipients reported that less than half of the participants were informed about ICD-related complications, restrictions related to driving, and end-of-life deactivation discussions. Additionally, a gender disparity emerged, with women being less involved in the decision-making process and less optimally informed [4].

Thus, it is imperative to investigate and understand how physicians effectively convey information to patients regarding critical medical treatment decisions—in this case, the appropriate uptake of CIEDs, specifically ICDs or CRT. For this study, the conversations between electrophysiologists and patients were targeted in order to (1) obtain objective data on the interaction between the electrophysiologist and the patient, (2) understand the framework of these conversations, and (3) identify innovative solutions through examination of these interactions. This research seeks to bridge existing gaps in patient education and communication to enhance patient understanding and involvement in decisions regarding CIEDs.

## Methods

### Participants

This interview and survey-based study was conducted across four countries: Australia, Spain, the UK, and the USA. The study recruited individuals who had previously been identified as requiring an ICD/CRT-D (hereafter referred to as “patient participants”), through country-specific recruiting agencies. Participants were recruited from healthcare databases in each market with the assistance of professional recruiters. All participants volunteered to join the study and received compensation for their time. They underwent a rigorous screening process, which included online surveys and phone calls, to ensure they met the study’s eligibility criteria. Additionally, the study included nine cardiac electrophysiologists who served as study physicians, with six based in the USA and one in each of the other three countries. All participants provided informed consent before participating in surveys and interviews. IRB approval was not obtained as the study did not involve the collection of personal health information, diagnostic procedures, or medical interventions.

### Study design

The study was conducted through a four-stage process: pre-conversation survey, patient participant-physician conversation, post-conversation survey, and follow-up interview. Research activities were organized by a third-party research organization (Ipsos, Paris, France).

#### Pre-conversation survey

Before engaging in the patient participant-physician conversations, both parties completed a pre-conversation survey. The patient survey aimed to establish prior experiences with the patient participants’ own physicians, gauge participants’ expectations, and estimate the time patient participants spent discussing the device implant with their own physicians prior to meeting with study physicians. 81.5% of the initial visits with the patients’ own physicians were conducted with a cardiac specialist. Patient willingness to undergo implant as well as several metrics to gauge patients’ satisfaction with physician conversations were also recorded and assessed using rating scales ranging from 1 (strongly disagree) to 7 (strongly agree). The physician survey assessed physician practice information, patient volume, and also collected information on typical conversation time/structure.

#### Patient participant-physician conversation

Prior to the conversation, study physicians were provided a profile containing pertinent information about the patient participants. These profiles included patient demographics, information about the patients’ cardiac conditions, and the patients’ decision or inclination regarding device implantation. Each study physician was matched with two to four patient participants from their respective country, and a 30-min video call was performed for each physician–patient participant pair.

During these video calls, study physicians were granted the freedom to conduct conversations about defibrillator devices (ICDs and CRT-Ds) in the manner consistent with how they would engage with one of their own patients. These interactions and conversations were observed and recorded for subsequent analysis. The identities and names of both the physicians and the patient participants were anonymized.

#### Post-conversation survey

Following the conversation, each study physician and patient participant completed a post-conversation survey. The purpose of this survey was to elicit feedback from participants regarding their impression of the conversation and gauge their overall satisfaction with the experience.

#### Follow-up interview

To expand on insights gathered from the post-conversation survey, follow-up 45-min interviews were conducted by the third-party research organization with each participant after their conversation with the study physician. The main objective of these interviews was to gain additional insights into participants’ expectations, experiences, and feedback regarding the conversation.

### Data analysis

To gain additional insights into the patient participant-study physician interaction, an in-depth analysis of surveys and conversation videos was conducted. The surveys were analyzed quantitatively using descriptive statistics to summarize the feedback elicited. The videos were analyzed both qualitatively and quantitatively, with a focus on identifying key themes and patterns in how study physicians conducted the conversation and how patient participants responded. To aid in this process, the researchers utilized Big Sofa, an AI-enabled video analytics technology (Big Sofa Technologies Group Limited, London, UK) [7]. This software is designed to conduct an intricate analysis of video content, with an array of advanced features such as automatic transcription, facial/motion recognition, and sentiment analysis. The algorithms applied in these technologies have been trained on extensive datasets, with the results cross-verified via manual analysis to ensure the accuracy and reliability.

The automatic transcription feature is capable of converting speech in the video data into text, thereby allowing a deeper analysis of the spoken content. The facial/motion recognition tool is designed to identify individuals’ expressions and body movements in the video, making it possible to track individual participation and reactions throughout the conversation. The sentiment analysis tool, on the other hand, is adept at understanding and interpreting the emotional tone conveyed in the dialogues, providing invaluable insights into the emotional dynamics of the physician/patient interaction.

These cutting-edge features collectively facilitated a streamlined extraction of insights from the video data. By leveraging this technology, our study was able to distill crucial information such as the themes explored during the conversations, the time allocated to each topic, specific actions taken by the physicians throughout the dialogue—such as the use of visual aids—and the terminology employed during the conversation. Additionally, we synthesized the tone and sentiment of the conversation to better understand the emotional dynamics at play. Following this, a comprehensive analysis was conducted that delved into the intricate dynamics of the physician/patient interaction, providing a nuanced understanding of this encounter.

Sentiment analysis is the process of identifying and categorizing the emotions expressed in a piece of text or conversation. These emotions are often categorized as positive, negative, or neutral. Sentiment analysis utilizes a machine learning model to analyze various linguistic features within text, such as word choice, sentence structure, and punctuation, to determine the overall emotional tone of the conversation on a spectrum from negative to positive. In the present study, Watson NLU Sentiment Analysis [8] was used to analyze the sentiment of patient-physician conversations. This involved transcribing the conversations into text format and then applying the sentiment analysis model to understand the emotional dynamics between patients and physicians.

Paired *t*-testing was conducted to identify any differences in patient participants’ feedback on their conversation with the study physicians versus their own physicians. A *p* < 0.05 was considered statistically significant. All analyses were performed using IBM SPSS Statistics 28.0 (IBM SPSS Inc., Chicago, IL, USA).

## Results

### Baseline characteristics of participants

A total of 27 patient participants from four countries participated in the study. All patient participants had received a diagnosis indicating the need for ICD or CRT-D but had decided not to undergo implantation. The age of the patient participants ranged from 30 to 70 years with a mean age of 51 years (Table 1). Over 60% of the patient participants were between the ages of 40–59. The gender distribution of the patient participants was balanced, with 51.9% of the participants being female. Most patient participants (85%) had been recommended ICD/CRT-D implantation by their own physicians within the 12 months leading up to their participation in the study. Before engaging in the conversation with the study physicians, the average willingness of the patient participants to undergo ICD/CRT-D implant was 4.4 on a scale ranging from 1 to 7. This measurement provided a baseline for assessing changes in willingness following structured conversations with study physicians.

**Table 1:** Baseline characteristics of patient participants

Patient characteristic	*N* = 27 patient participants
Age (years)	51.1 ± 10.1
Female sex, no. (%)	14 (51.9%)
Geographic distribution	
USA	18 (66.7%)
UK	3 (11.1%)
Spain	3 (11.1%)
Australia	3 (11.1%)
Highest education level	
High school	5 (18.5%)
Associate degree	2 (7.4%)
Bachelor’s degree	14 (51.9%)
Master’s degree	6 (22.2%)
Household income level (local currency has been used in the categorization)*	
Lower	6 (22.2%)
Medium	9 (33.3%)
Higher	12 (44.4%)
Insurance type	
Private	14 (51.9%)
Public	13 (48.1%)
Time since been talked by a physician to get a device implanted in heart	
Less than 6 months	11 (40.7%)
6 months to 12 months	10 (37.0%)
19 to 23 months	3 (11.1%)
2 to 5 years	3 (11.1%)
Willingness to get an implanted device (on a scale from 1 to 7, 1 being very unwilling and 7 being very willing)	
1	0 (0%)
2	3 (11.1%)
3	3 (11.1%)
4	7 (25.9%)
5	10 (37.0%)
6	2 (7.4%)
7	2 (7.4%)

^*^Lower: < $60 K in the USA, < 20 K€ in Spain, < A$60 K in Australia, < £30 K in the UK; Medium: $60 K-$100 K in the USA, 40 K€-80 K€ in Spain, A$60 K-A$120 K in Australia, £30 K-£60 K in the UK; Higher: > $100 K in the USA, > 80€ in Spain, > A$120 K in Australia, > £60 K in the UK

Age is represented by mean ± SD. Categorical variables are represented by absolute number of participants and (percentage)

### Baseline demographics of study physicians

Nine cardiac electrophysiologists served as study physicians, with six based in the USA and one in each of the other three countries (Table 2). Of the 9 study physicians, 7 (77.8%) were female and over half (55.6%) had been practicing 10–20 years, while one-third had been practicing for > 20 years.

**Table 2:** Study physician characteristics

Physician characteristic	*N* = 9 study physicians
Female sex, no. (%)	7 (77.8%)
Years of practice	
< 5 years	0 (0%)
5–10 years	1 (11.1%)
10–20 years	5 (55.6%)
20 + years	3 (33.3%)
Geographic distribution	
USA	6 (66.7%)
UK	1 (11.1%)
Spain	1 (11.1%)
Australia	1 (11.1%)

Categorical variables are represented by absolute number of participants and (percentage)

### Conversation structure

Although study physicians were granted autonomy to conduct the conversation in the manner they would with one of their own patients, a consistent structure was observed in conversations, irrespective of physicians’ country. The general structure and flow of the conversation involved several key components, commencing with introductions and review of medical history, followed by explanations of the device, undergoing the implantation, and concluded with the long-term consequences of living with the device.

**Fig. 1:**
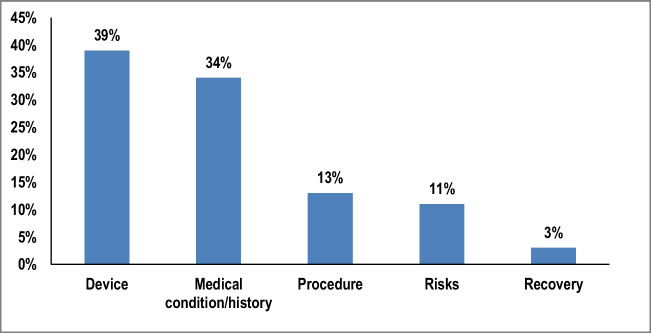
Share of time spent discussing each topic. Bars depict average % of time spent on topics across all conversations

The general flow of the conversations was as follows—during the introductions, the study physicians confirmed the patient participants’ medical history and discussed their heart condition and associated risks, such as sudden cardiac death. They then explained the ICD/CRT-D device and its purpose as a defibrillator. The physicians typically highlighted the value of having the device given the patient’s medical history, and the surgery itself was frequently described as a low-risk procedure (70.4% of the patient conversations in this study). The majority of the physicians (7 out of 9 in this study) showed the patient the approximate size and location of the incision and described the lead insertion through a vein into the heart. Patients were also shown the device and its size through different visuals (78% through hand demonstrations, 19% through an actual device, and 3% through drawing of heart). The physicians explained the recovery period after surgery and discussed the risk of infection, both post-operatively and over time. They also discussed the risk of device malfunction, including shocks, the risk of broken leads and their replacement, and the battery replacement timeline. Analysis of the conversations found that study physicians spent the most time discussing the device (39%) and medical condition/history with patient participants (34%, Fig. 1).

AI-driven analysis of the conversation structure revealed that study patients expressed a generally positive sentiment [8] when discussing topics related to ICD/CRT-D devices, the procedure, and the recovery after the procedure with the study physicians. However, it was observed that the patients’ sentiment shifted more negatively when engaging in discussions about their medical history/condition and the associated risks of the procedure.

### Conversation feedback and attitude change

**Fig. 2:**
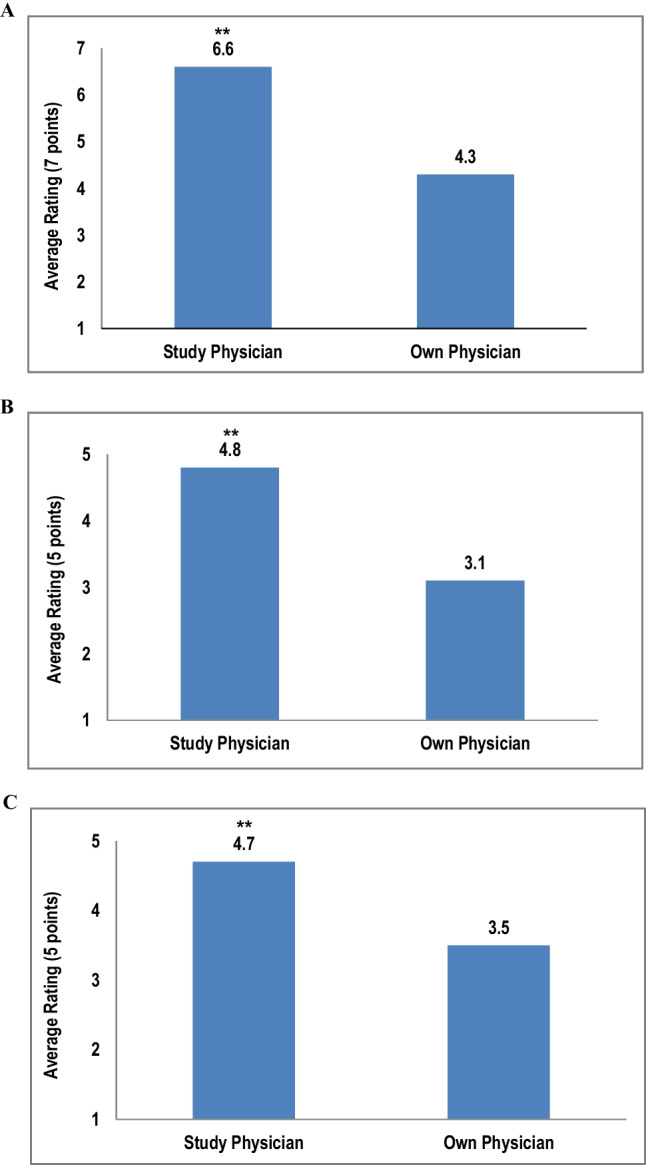
Patient participants’ rating on the quality of the conversation. Bar graphs depict average ratings provided by patient participants for the following survey questions: How satisfied were you with your experience discussing the surgery with the physician? (**A**); How well did the physicians explain the device and the surgery? (**B**); How well did the physician answer your questions about the device and surgery? (**C**). Ratings based upon scale as noted for each graph. ** = *p* < 0.001

Patient participants reported higher satisfaction with the study conversation compared to their own physicians before the study (*p* < 0.001, Fig. 2A). Patient participants also indicated that the study physicians explained the device and surgery more effectively than their own physicians, qualifying the explanations with terms such as, “thorough, in-depth, detailed, and informative” (p < 0.001, Fig. 2B). Participants reported positive feedback regarding the study physicians’ ability to answer their questions (*p* < 0.001, Fig. 2C).

**Fig. 3:**
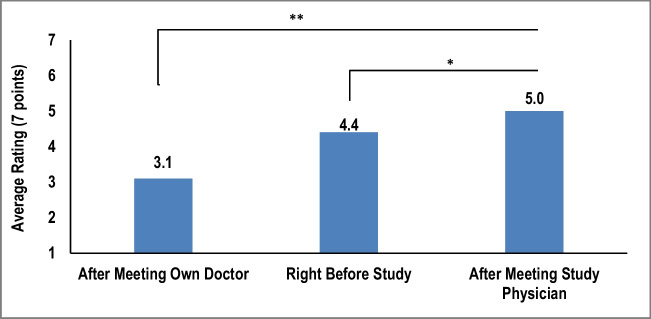
Patient participants’ attitude change after the study. Survey results reflecting to what extend the patient participants were willing to move forward with getting a new ICD/CRT device. The exact survey question asked: To what extent are you willing to move forward with getting a device that could shock your heart? Bars represent average rating on a 7 point scale. * = *p* < 0.05; ** = *p* < 0.001

Patient participants were significantly more willing to undergo clinically indicated ICD/CRT-D implantation after engaging in conversations with the study physicians compared to their own physicians and pre-conversation surveys conducted just prior to the patient participant-study physician interaction (mean scores: 5.0, 3.1, and 4.4 out of 7, respectively; *p* < 0.001, Fig. 3). Education level and household income of patient participants did not have a significant impact on willingness to undergo implantation (both *p* > 0.05).

### Knowledge sharing

Survey data revealed that study physicians did a significantly better job in maintaining patient participants attention, making the information easy to understand, and giving sufficient information regarding the device (*p* < 0.05 or p < 0.001, Table 3).

**Table 3:** Patient participants’ ratings about conversation details

Agreement rating of statements about the conversation (7 points) 1, strongly disagree; 7, strongly agree		Own physician	Study physician
It was **hard for me to pay attention** to the physician during the conversation		3.7	1.5**
During our conversation about a heart device that could shock my heart, the information I talked about with the physician was **hard to understand**		4.0	1.3**
I **need some time to think about a heart device** that could shock my heart after the discussion with the physician		6.2	4.6**
During the conversation about a heart device that could shock my heart, the physician **gave me a chance to express my opinions** about getting a heart device		4.9	6.1*
During the conversation about a heart device that could shock my heart, the Study Physician **gave me a chance to ask for as many questions as I needed** about a heart device		5.1	6.3*
During the conversation, the physician **gave me enough information** about a heart device that could shock my heart		4.2	6.0**
To what extent was the Physician?	Trustworthy	6.4	6.3
	Dependable	6.4	6.3
	Honest	6.5	6.4
	Reliable	6.3	6.3
	Sincere	6.1	6.5
	Easy to understand	5.7	6.6*

Paired *T*-test: *P < 0.05, **P < 0.001

**Fig. 4:**
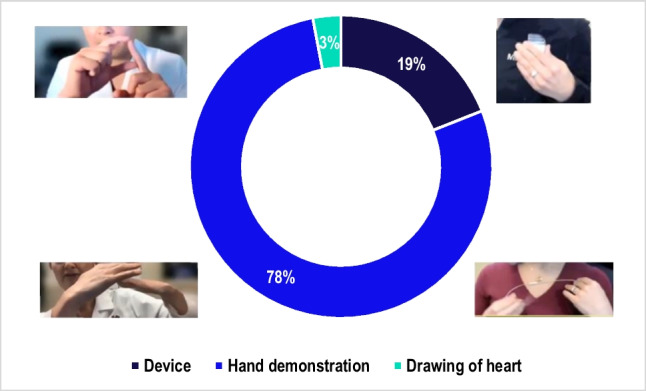
The use of visual aid types. Breakdown of visual aid types used by study physicians in patient participant-study physician conversations. Results are reflective of 194 uses of visual aids across 27 conversations

The use of visual aids during the conversation was utilized frequently by physicians as a knowledge sharing tool. Study physicians used physical devices, hand demonstrations, or drawings to explain the surgical procedures and device workings. Either real devices or leads and/or hand demonstrations were used to illustrate the size and placement of the device on the chest and how the leads are placed into the vein or heart. Visual aids, such as hand-drawn or digital images, were also used to show the heart and veins and what they looked like with leads inside them. Hand demonstrations paired with metaphors in the description worked well when the actual device was absent and was the most frequently used visual aid by study physicians (78% of visual aids, Fig. 4). Study patients expressed a preference for these visual demonstrations as learning resources rather than text-heavy materials or pamphlets. Online resources such as the Colorado Program for Patient Centered Decisions were recognized as helpful by the majority of patients but statistics aiming to emphasize the benefits of ICD negatively impacted patients’ sentiments regarding the procedure. The statistic “7 lives saved over 5 years by having an ICD among a group of 100 patients” seen in the Colorado Program for Patient Centered Decisions pamphlet, diminished the perceived effectiveness of the devices for some participants.

### Bedside manner

The AI-driven video analysis suggested that eye contact and active listening, as indicated by study physician head nods, contributed to patient engagement. For the statement “hard to pay attention during the conversation,” patient participants rated the conversations with study physicians favorably versus their own physicians (1.5 vs. 3.7/7; *p* < 0.001, with lower numbers indicating a more favorable rating). Additionally, study physicians were rated higher in allowing patient participants to express their opinions regarding getting a device (6.1 vs 4.9, *p* < 0.05, Table 3). Survey data indicated that study physicians were deemed empathetic, approachable and respectful.

### Answering questions efficiently

Study physicians were rated significantly higher than patient participants’ own physicians in both offering opportunities to ask questions and in answering those questions. Specifically, patient participants felt they were able to ask as many questions as needed during the study conversation versus the conversation they had with their own physician (6.3 vs. 5.1 out of 7; *p* < 0.05, Table 3). Also, as noted above, study physicians were considered to have answered those questions better than participants’ own physicians (Fig. 3c).

There were certain questions that recurred across patient conversations, i.e., the top frequently asked questions (FAQs) that were identified in the analysis (Table 4). These included the following: (1) how the cardiac device works, (2) if the device prevents a patient’s condition from worsening, (3) if the device affects or improves day-to-day activities, (4) if the cardiac device improves the patient’s symptoms, and (5) does the device require replacement. Patients were noted to be curious about both technical aspects of the device but also their quality of life after surgery. Survey data revealed that participants were relieved to hear that the device is monitored closely after implant and that quality of life can be improved.

**Table 4:** Frequently asked questions from patient participants

*How a heart device will work*
*If a heart device will help stop my heart condition from getting worse*
*If a heart device will make my day-to-day activities better*
*If a heart device will improve my symptoms / make me feel better*
*If a heart device ever needs to be replaced*

### Time spent

The average length of the study conversation was 28.4 ± 6.8 min, which was significantly longer than the patient participants’ average self-reported length of conversation with their own physicians of 19.9 ± 5.2 min (*p* < 0.001).

## Discussion

This study suggests that there is a “recipe for success” in the shared decision-making conversations related to device implantation which can significantly improve a patient’s engagement and satisfaction with the discussion as well as enhance appropriate, or informed, acceptance of ICD or CRT implantation. This study highlights that there are several key elements that collectively form the foundation for a productive interaction between the individual patient and the electrophysiologist, leading to a high-quality, successful conversation as indicated by patient sentiment and satisfaction:

1. A consistency in the flow and thoroughness in the approach to the conversation.
2. Effective communication of knowledge.
3. The show of empathy and appropriate bedside manner.
4. The importance of a dialogue and a forum for answering questions.
5. Allowing sufficient time for these interactions.

Study physicians followed a consistent routine in the conversational structure, across all countries, maintaining consistency in their approach. This standardized routine typically included a self-introduction and inquiry into the patient’s medical conditions. Study physicians provided a thorough introduction to the device, emphasizing the device’s life-saving potential. The procedure was also discussed in detail as well as the risks surrounding the device and the procedure. Finally, study physicians touched on the practicalities of living with the device and the compatibility of the device with aspects of everyday activities, specific areas where recent studies have demonstrated gaps in the information provided to patients [4].

Study physicians’ knowledge and information sharing was noted and recognized by the study patients. Compared with their regular physicians, study physicians performed significantly better in engaging the patient during the consultation, making the conversation easier to understand, and providing straightforward and easily comprehensible information to patients, thereby improving overall communication. Study physicians promoted a patient-centered approach by encouraging patients to ask questions that were needed to achieve patient comfort. Visual aids were found to be beneficial and shown to facilitate the conversation by contributing to improved patient understanding and satisfaction.

Patient preference for visual aids has been well described in the literature. Prior studies have shown that pictograms improve medication understanding and adherence even in low-literacy patients [9, 10]. A recent study in patients undergoing planned surgery showed that a visual aid in the informed consent process increased post-operative knowledge and provided greater benefit than self-directed research [9]. Showcasing the ICD generator and leads during the conversation and demonstrating the implantation procedure to the patient with a visual aid improved willingness to accept a cardiac device. However, in this study, there were some limitations with the learning resources that were used for shared decision-making of ICD implantation. Even though high-quality online sources of information, such as the Colorado Program for Patient Centered Decisions [11] were recognized as helpful, the presentation of statistics within these resources had the potential to negatively impact patients’ perception of the benefit of CIEDs. The International Patient Decision Aid Standards (IPDAS) quality criteria emphasize that decision aids are written in plain language that can be understood by the majority of patients (grade 8 or less) and that these tools help patients understand information through modalities other than reading [12]. Accordingly, in this study, visual demonstrations were preferred over text-heavy materials as learning resources. Overall, however, it is well-established that patients exposed to decision aids feel more knowledgeable and clearer about their values with more participation in the process and more accurate expectations of risk/benefit [13].

Physicians’ eye contact and active listening as identified by AI video analysis (signaled by specific expressions and movements such as a head nod) were bedside mannerisms that were key to maintaining patient engagement and comfort and patients reciprocated with improved attention. This environment was conducive to patients’ comfort in asking questions and had a positive impact on the study patient-physician interaction. There were certain questions about the device and its effects on the heart and the patient’s quality of life that were common themes in the discussions with the study physicians, i.e., FAQs (frequently asked questions). Study physicians were more effective than participants’ own physicians in both offering opportunities to ask questions and in answering those FAQs (see Table 4). This translated to participants expressing a higher degree of satisfaction with the conversation with study physicians.

Data from this study regarding the time spent by the study physicians with the patients indicate that for an effective conversation for such a critical topic as implantation of an ICD/CRT-D, at least 20 min should be set aside with the ideal duration being closer to 25 min. However, the reality in clinical practice is that many of these conversations occur in a much more limited time frame.

A truly novel aspect of this study was the use of artificial intelligence post hoc to extract valuable insights from the video data pertaining to patient behaviors and responses during the conversation [14]. Using this cutting-edge AI-enabled video analytics technology, individuals’ expressions and body movements were studied, the spoken content was extensively analyzed, and patient sentiments were captured. We are on the cusp of an explosion of the use of artificial intelligence in medicine, particularly in the analysis of patient behavior, with the aim of driving improvements in the provision of care. The information garnered through the AI analysis highlights the utility of this tool and its potential to transform the way we communicate with and deliver care for patients. For example, in this study, the application of AI revealed that patient sentiment shifted more negatively when discussing topics like their medical history/condition and the risks of the procedure. Discussing risks related to device implantation should not be avoided; instead, this highlights an opportunity for physicians to place more emphasis or dedicate more time to these specific aspects during the conversation to alleviate concerns. The ability of AI to identify the precise elements that could be targeted by physicians can improve the patient experience and facilitate optimal shared decision-making.

From a clinical perspective, an improved acceptance of guideline-directed device therapy positively impacts patient-related outcomes in the long term. Data strongly support an all-cause mortality benefit of ICDs in patients with ischemic cardiomyopathy and meta-analyses have supported their use in non-ischemic patients especially with indications for cardiac resynchronization therapy (CRT) [15–18]. CRT is an established treatment option that significantly improves quality of life as well as mortality. In large clinical trials, the reduction in all-cause mortality in patients with CRT versus optimal medical therapy ranges from 25 to 36% [19–21]. Despite the proven benefits of this subgroup of CIEDs, ICDs as well as CRT therapy remain markedly underutilized in many countries [22, 23]. This underutilization is partially driven by patient preference, i.e., refusal to undergo the treatment. A large study of a claims database found that 17% of patients eligible for an ICD who did not receive one had refused the device [24]. Prior studies have identified that patients who refuse ICDs have common patient-related barriers including lack of insight regarding their personal risk, perceived strength of the recommendation by their physician, concerns over malfunction and surgical risk, and inaccurate perception of lifestyle changes [25]. Shared decision-making is a process through which care is tailored to the values and informed preferences of the patient based on the clinical evidence and expertise of the clinician. Improved patient understanding and participation as a result of a robust, high-quality dialogue can tackle many of these barriers. The objective is not increased utilization per se but rather ensuring that other factors such as ineffective communication do not interfere with an informed decision by the patient to accept a beneficial therapy.

Overall, this comprehensive analysis provides invaluable insights into the dynamics of physician/patient conversations and the ways in which they can be improved to better meet patient needs. The insights from this study were both qualitative and quantitative. Informed consent is an ethical imperative and requires shared decision-making through education and engagement of patients. This study provides a roadmap for a successful patient-physician conversation.

### Limitations

Limitations of the study include the small sample size which could affect the significance and generalizability of the results. Additionally, the small sample size may not be reflective of the broader population; for example, over 70% of participants had a bachelor’s or master’s degree, which may affect conclusions that involve health literacy. All interviews were conducted via telemedicine which could influence the perceived interaction and sentiments of the conversation and could have provided disparate results versus an in-person discussion. Additionally, qualitative and quantitative data collected on the patient participants’ conversation with their own physician is based on the patients’ recollection of this conversation (for the vast majority, this had occurred within the last year); this recall bias could skew some of the results and observations made in this study.

## Conclusions

It is imperative that electrophysiologists undertaking informed consent discussions regarding ICDs/CRT devices are familiar with the “keys to success” for an effective dialogue with the patient. A comprehensive understanding of FAQs related to these devices can maximize the shared decision-making conversation. The key elements identified by this study using novel AI-enabled video analysis could be incorporated into tools and user-friendly educational materials designed to facilitate these crucial conversations that have potentially life-altering implications. Consequently, if consistent with the patient’s values and informed preferences, improving the willingness of patients to accept a clinically indicated ICD or CRT system will, in turn, positively impact patient-related outcomes in heart failure and SCD given the well-established benefits.
